# Polycomb repressive complex 2 facilitates the nuclear export of the influenza viral genome through the interaction with M1

**DOI:** 10.1038/srep33608

**Published:** 2016-09-20

**Authors:** Masamitsu N. Asaka, Atsushi Kawaguchi, Yuri Sakai, Kotaro Mori, Kyosuke Nagata

**Affiliations:** 1 Department of Infection Biology, Faculty of Medicine and Graduate School of Comprehensive Human Sciences, University of Tsukuba, Tsukuba, Japan; 2 Ph.D. Program in Human Biology, School of Integrative and Global Majors, University of Tsukuba, Tsukuba, Japan

## Abstract

The organization of nuclear domains is crucial for biological events including virus infection. Newly synthesized influenza viral genome forms viral ribonucleoprotein (vRNP) complexes and is exported from the nucleus to the cytoplasm through a CRM1-dependent pathway mediated by viral proteins M1 and NS2. However, the spatio-temporal regulation of the progeny vRNP in the nucleus is still unclear. Here we found that polycomb repressive complex 2 (PRC2), which contains a methyltransferase subunit EZH2 and catalyzes histone H3K27me3 for the formation of facultative heterochromatin, is a positive factor for the virus production. Depletion of PRC2 complex showed the nuclear accumulation of vRNP and the reduction of M1-vRNP complex formation. We also found that PRC2 complex directly binds to M1, and facilitates the interaction of M1 with vRNP. In conclusion, we propose that the progeny vRNP could be recruited to facultative heterochromatin and assembled into the export complex mediated by PRC2 complex.

The genome of influenza A virus consists of eight-segmented and single-stranded RNAs of negative polarity (vRNA). vRNA forms viral ribonucleoprotein complexes (vRNP) with viral RNA-dependent RNA polymerases and nucleoprotein (NP). After the nuclear import of incoming vRNP, nuclear structures and functions are remodeled possibly for the replication and transcription of vRNA, and post-replicational processes^1^.

The nucleus is highly compartmentalized into chromosome territories and numerous distinct nuclear compartments such as nucleolus, promyelocytic leulemia nuclear body (PML NB), spliceosome, and so on. Chromatin forms either a condensed and transcriptionally inactive structure called heterochromatin or a relaxed and transcriptionally active structure called euchromatin. Heterochromatin is classified into two groups based on histone modifications. One is constitutive heterochromatin (cHC) marked by histone H3 lysine 9 trimethylation (H3K9me3) mediated by a variety of factors including, RIZ1, SUV39H1, SUV39H2, SETDB, and so on^2^. The other is facultative heterochromatin (fHC) marked by histone H3 lysine 27 trimethylation (H3K27me3) mediated by polycomb repressive complex 2 (PRC2)^3^ and histone H2A lysine 119 (H2AK119Ub) ubiquitination mediated by polycomb repressive complex 1 (PRC1)^4^.

After transport of incoming influenza virus vRNP into the nucleus, viral polymerases interact with cellular RNA polymerase II machineries for the viral transcription at euchromatin^5^,^6^,^7^,^8^. vRNP also interacts with heterochromatin through the histone tails containing H3K9me3 and H3K27me3^9^,^10^,^11^,^12^. However, the functional significance of heterochromatin localization of vRNP is unknown. After viral genome replication, newly synthesized vRNA is assembled into vRNP, followed by export from the nucleus to the cytoplasm mediated by the CRM1-dependent pathway^13^,^14^,^15^. A current model of the nuclear export of vRNP suggests that M1 directly binds to vRNP^16^,^17^, and M1 is then linked to CRM1 by NS2 through its nuclear export signal (NES)^15^,^18^. It has been suggested that heterochromatin functions as a scaffold to form vRNP export complexes due to the localization of vRNP export complexes at heterochromatin^19^. It is also reported that M1 and NS2 are localized at PML NB with vRNA in cells treated with a potent CRM1 inhibitor, leptomycin B^20^. However, the detail of the spatio-temporal dynamics of the progeny vRNP in the nucleus is unclear.

Here we found that a punctate localization pattern of EZH2, a catalytic subunit of PRC2 complex, becomes dispersed after influenza virus infection. vRNP was found to interact with PRC2 complex in infected cells. EZH2, EED, and SUZ12 are required for a minimal methyltransferase activity of PRC2 complex^21^,^22^. EED recognizes histone H3K27me3 to recruit PRC2 complex onto fHC to maintain the methylation level^23^. SUZ12 binds to EZH2 through EED and stimulates the methyltransferase activity^21^. Knockdown (KD) analyses indicated that EZH2 stimulates the vRNP export in a methyltransferase activity-independent manner. Further, we showed that PRC2 complex facilitates the association of M1 with vRNP. Taking these findings together, we propose that PRC2 complex promotes the nuclear export of vRNP by recruiting M1 onto vRNP.

## Results

### PRC2 complex is a positive factor for the virus production

**Figure 1:**
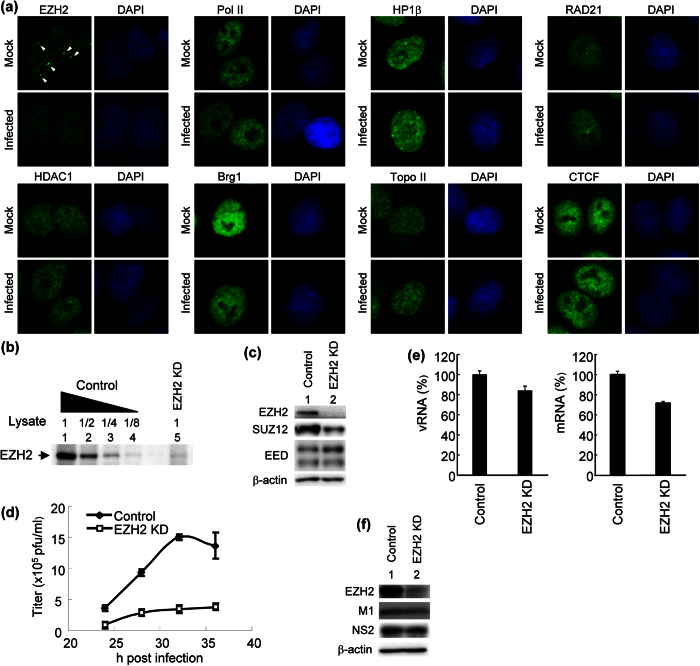
PRC2 complex is involved in the viral life cycle. (**a**) Remodeling of nuclear compartments by influenza virus infection. At 6 h post infection, mock-infected (upper panel) or infected (lower panel) HeLa cells were subjected to indirect immunofluorescence assays with anti-EZH2, anti-Pol II, anti-HP1β, anti-RAD21, anti-HDAC1, anti-Brg1, anti-Topo II, and anti-CTCF antibodies, respectively (green). Nuclei were counter-stained with DAPI (blue). Punctate foci of EZH2 are indicated by arrowheads. (**b**) Knockdown efficiency of EZH2. HeLa cells were transfected with either control siRNA or siRNA against EZH2. At 60 h post transfection, 1 × 10^5^ cells (lane 1), 5 × 10^4^ cells (lane 2), 2.5 × 10^4^ cells (lane 3), 1.25 × 10^4^ cells (lane 4) of control cells, and 1 × 10^5^ cells of EZH2 KD cells (lane 5) were subjected western blotting with anti-EZH2 antibody. The original blot is presented in Supplementary Fig. 1. (**c**) The amounts of PRC2 complex components in EZH2 KD cells. Control or EZH2 KD cells were subjected to western blotting with anti-EZH2, anti-SUZ12, anti-EED, and anti-β-actin antibodies. The original blots are presented in Supplementary Fig. 2. (**d**) Production of infectious virions. At 60 h post transfection, control and EZH2 KD cells were infected with influenza virus at MOI of 0.3. The culture supernatants were collected at 24, 28, 32, and 36 h post infection, and subjected to plaque assays. The average titers and standard deviations determined from three independent experiments are shown. (**e**) Expression levels of vRNA and mRNA in EZH2 KD cells. Total RNAs prepared from control and EZH2 KD cells at 6 h post infection were subjected to the quantitative real-time PCR. The amounts of vRNA and mRNA of segment 5 in control cells were indicated as 100%, respectively. The average expression levels and standard deviations determined from three independent experiments are shown. (**f**) Expression levels of M1 and NS2 in control and EZH2 KD cells. At 6 h post infection, control and EZH2 KD cells were subjected to SDS-PAGE, and carried out western blotting with anti-EZH2, anti-M1, anti-NS2, and anti-β-actin antibodies. The original blots are presented in Supplementary Fig. 3.

To analyze the effect of the virus infection on the nuclear compartments, we carried out indirect immunofluorescence assays with either mock-infected or infected cells using antibodies against cellular factors related to the nuclear structures such as euchormatin, heterochromatin and nuclear matrix (Fig. 1a). We found that punctate foci of EZH2 becomes dispersed through the nucleus in response to the infection, although the function of EZH2 foci is unclear^24^. In contrast to the case for EZH2, we did not observe localization changes for other proteins. EZH2 is a catalytic subunit of PRC2 complex methyltransferase. It is known that PRC2 complex induces the fHC formation through the methyltransferase activity of histone H3K27me3 and represses cellular gene expression. To examine whether PRC2 complex is involved in a process of progeny virion production, we examined the effect of EZH2 KD on the virus titer. The expression level of EZH2 in KD cells decreased to approximately 15% of that in control cells at 72 h post transfection (Fig. 1b and Supplementary Fig. 1). EZH2 KD reduced the amounts of SUZ12 due to the degradation as previously reported (Fig. 1c and Supplementary Fig. 2)^25^. The amount of infectious progeny virions produced from EZH2 KD cells was reduced to approximately 30% of that produced from control cells (Fig. 1d). Thus, we concluded that PRC2 complex stimulates the production of infectious viruses.

EZH2 and other PRC2 complex components are predominantly localized in the nucleus (Fig. 1a). We assumed that PRC2 complex affects the viral genome functions in the nucleus, such as the viral genome replication, viral transcription, and nuclear export of vRNP. Thus, we examined the amounts of vRNA and viral mRNA in EZH2 KD cells at 6 h post infection. We found that the expression levels of vRNA and viral mRNA in EZH2 KD cells were unchanged compared with those in control cells (Fig. 1e). In addition, the expression levels of viral proteins were not affected by EZH2 KD (Fig. 1f and Supplementary Fig. 3). Therefore, it is possible that PRC2 complex is not involved in the replication and transcription of the viral genome.

### PRC2 complex is required for the nuclear export of vRNP

**Figure 2:**
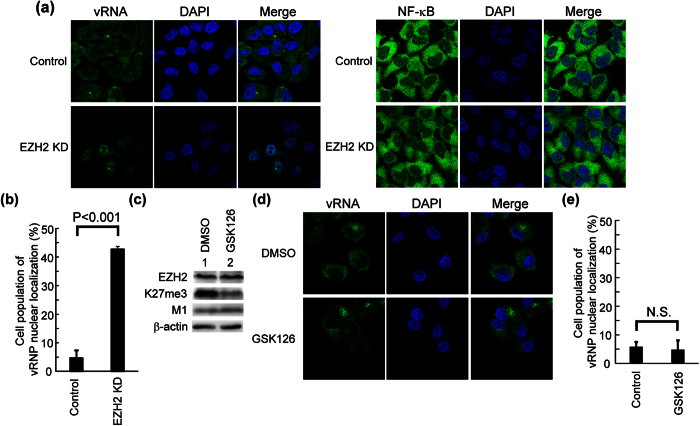
PRC2 complex is involved in the nuclear export of vRNP. (**a**,**b**) Intracellular localization of the viral genome in control and EZH2 KD cells. At 60 h post transfection of siRNA, control and EZH2 KD cells were infected with influenza virus at MOI of 5 (left panels). At 6 h post infection, FISH assays were carried out with an RNA probe complementary to segment 1 vRNA (green). Nuclei were counter-stained with DAPI (blue). The average number of the cells, in which more than 60% of vRNA shows nuclear localization pattern, was determined from three independent experiments (panel b; *n* > 100). To visualize endogenous NF-κB, control and EZH2 KD cells were subjected to indirect immunofluorescence assays with anti-NF-κB antibody (right panels). The level of significance was determined by Student’s *t* test. (**c**,**d,e**) HeLa cells were treated with either DMSO or 500 nM of GSK126 for 60 h. The cells were infected with influenza virus at MOI of 5. At 6 h post infection, cells were subjected to western blotting with anti-EZH2, anti-histone H3K27me3, anti-M1, and anti-β-actin antibodies (panel c). The original blots are presented in Supplementary Fig. 4. To examine the intracellular localization of vRNA, FISH assays were carried out with the RNA probe complementary to segment 1 vRNA (panel d, green). Nuclei were counter-stained with DAPI (blue). The average number of cells, in which more than 60% of vRNP shows the nuclear localization pattern, was determined from three independent experiments (panel e; *n* > 100). The level of significance was determined by Student’s *t* test. N. S., not significant (P > 0.05).

Next, we examined the nuclear export of vRNP by fluorescent *in situ* hybridization (FISH) assays. In control cells, more than 90% of cells showed the cytoplasmic localization pattern of vRNA at 6 h post infection (Fig. 2a,b). On the other hand, approximately 43% of EZH2 KD cells exhibited the nuclear localization of vRNA, suggesting that PRC2 complex is required for the nuclear export of vRNP (Fig. 2a,b). NF-κB is a nucleocytoplasmic shuttling protein mediated by CRM1, and showed the nuclear localization by inhibiting CRM1^26^. In contrast to vRNP, NF-κB was successfully exported to the cytoplasm in control and EZH2 KD cells, suggesting that EZH2 KD does not impair the CRM1 function (Fig. 2a). The expression levels of M1 and NS2, which are viral proteins and involved in the nuclear export of vRNP, were not different between control and EZH2 KD cells (Fig. 1f and Supplementary Fig. 3).

It has been reported that CRM1 and other viral proteins were recruited to heterochromatin, and heterochromatin functions as a scaffold to assemble the export complex of vRNP^19^. Thus, it is possible that EZH2 KD disrupts the fHC structure, thereby inhibiting the vRNP export. Hence, we next examined whether the methyltransferase activity of PRC2 complex is required for the vRNP export using GSK126, a potent methyltransferase inhibitor of EZH2^27^. The histone H3K27me3 level was decreased to 25% in GSK126-treated cells (Fig. 2c and Supplementary Fig. 4), but we found that vRNP was successfully exported to the cytoplasm in GSK126-treated cells at 6 h post infection (Fig. 2d,e). Thus, it is likely that the nuclear accumulation of vRNP in EZH2 KD cells is not due to the reduction of the histone H3K27me3 level, and the methyltransferase activity of PRC2 complex is not involved in the nuclear export of vRNP.

### PRC2 complex interacts with vRNP-M1 complex

**Figure 3:**
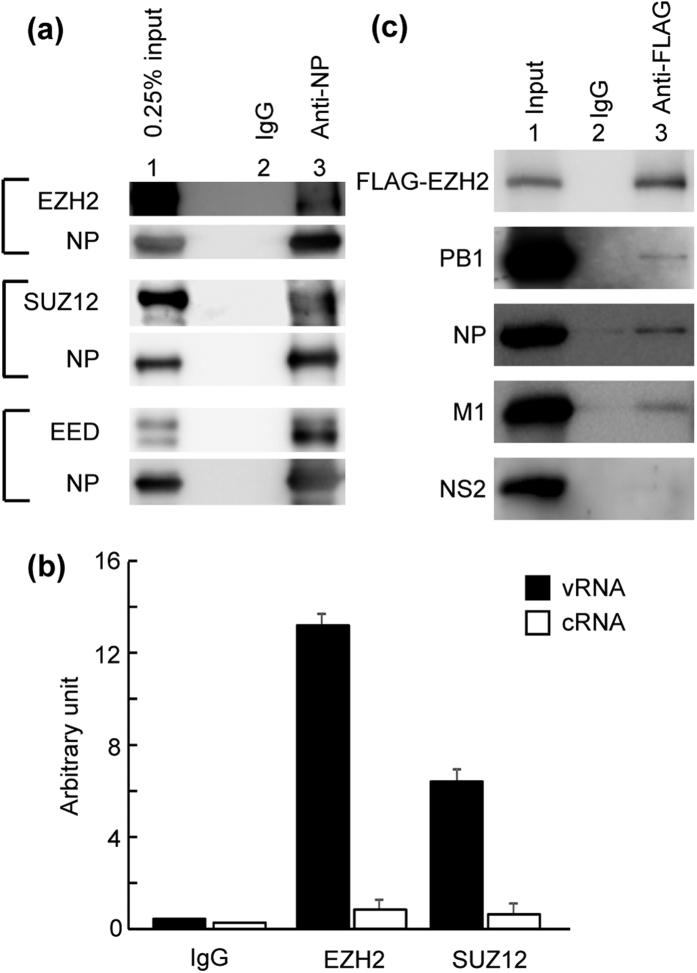
Interaction of PRC2 complex with vRNP complex in infected cells. (**a**) Interaction of vRNP with PRC2 complex. At 6 h post infection, infected cells were subjected to immunoprecipitation assays with either non-specific IgG (lane 2) or anti-NP (lane 3) antibody-conjugated protein A Sepharose. Co-immunoprecipitated proteins were separated by 10% of SDS-PAGE, and analyzed by western blotting with anti-EZH2, anti-EED, anti-SUZ12, and anti-NP antibodies. The original blots are presented in Supplementary Fig. 5. (**b**) Interaction of PRC2 complex with vRNA. At 6 h post infection, infected HeLa cells were subjected to immunoprecipitation assays with non-specific IgG, anti-EZH2, and anti-SUZ12 antibodies, respectively. The absolute amounts of precipitated RNAs were quantified by quantitative real-time PCR as descried in Methods. (**c**) Interaction of EZH2 with vRNP complex. HeLa cells were transfected with a plasmid expressing 3xFLAG-EZH2, and then infected with influenza virus at MOI of 5. At 6 h post infection, these cells were subjected to immunoprecipitation assays with either non-specific IgG (lane 2) or anti-FLAG (lane3) antibody-conjugated Sepharose. Co-immunoprecipitated proteins were separated by 7.5% or 12.5% of SDS-PAGE, and analyzed by western blotting with anti-FLAG, anti-PB1, anti-PB2, anti-NP, anti-M1, and anti-NS2 antibodies. The original blots are presented in Supplementary Fig. 6.

From the results shown in Fig. 2, it is possible that PRC2 complex binds to vRNP and stimulates the nuclear export of vRNP. To address this, we carried out immunoprecipitation assays with infected cell lysates using anti-NP antibody to detect the interaction of NP/vRNP with PRC2 complex. We found that not only EZH2 but also EED and SUZ12 were co-immunoprecipitated with vRNP (Fig. 3a and Supplementary Fig. 5). Further to examine whether these chromatin proteins interact with NP associated with viral RNAs, the cell lysates prepared from infected cells were subjected to immunoprecipitation assays with anti-EZH2 and anti-SUZ12 antibodies, respectively. The amounts of co-precipitated viral RNAs were quantitatively determined by quantitative RT-PCR. The amount of vRNA co-immunoprecipitated with endogenous EZH2 and SUZ12 was 17.31 ± 0.57 and 10.36 ± 0.64 times more than cRNA, respectively (Fig. 3b). These results indicate that PRC2 complex interacts with vRNP in infected cells. Next, we carried out immunoprecipitation assays with cell lysates prepared from infected HeLa cells expressing 3xFLAG-EZH2. We found that viral polymerases, NP, and M1 were co-immunoprecipitated with 3xFLAG-EZH2, but NS2 was not (Fig. 3c and Supplementary Fig. 6).

### PRC2 complex is required for the interaction between M1 and vRNP

**Figure 4:**
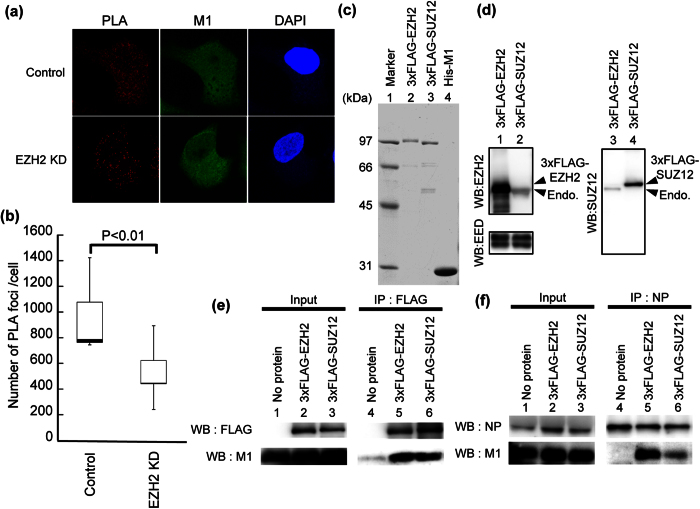
PRC2 complex interacts with M1, and facilitates the interaction between M1 and vRNP. (**a**,**b**) *in situ* PLA assays. At 60 h post transfection of siRNA, control and EZH2 KD cells were infected with influenza virus at MOI of 5. At 6 h post infection, cells were subjected to *in situ* PLA assays with rabbit anti-M1 and mouse anti-NP antibodies (red), then indirect immunofluorescence assays were carried out to detect the intracellular localization of M1 (green). Nuclei were counter-stained with DAPI (blue). The number of PLA signals was obtained from three-dimensional reconstruction images by IMARIS software (*n* = 11). The level of significance was determined by Student’s *t* test. (**c**) Purification of recombinant proteins. Purified 3xFLAG-EZH2, 3xFLAG-SUZ12, and His-M1 proteins were applied to 10% SDS-PAGE, and visualized by CBB staining. The original blot is presented in Supplementary Fig. 7. (**d**) Western blotting of purified proteins. Purified proteins were subjected to western blotting with anti-EZH2, anti-SUZ12, and anti-EED antibodies. The original blot is presented in Supplementary Fig. 8. (**e**) Interaction of PRC2 complex with M1. Recombinant His-M1 (12 pmol) was incubated without (lanes 1 and 4) or with either 8 pmol of 3xFLAG-EZH2 (lanes 2 and 5) or 8 pmol of 3xFLAG-SUZ12 (lanes 3 and 6) for 1 h. Then, the proteins were subjected to immunoprecipitation assays with anti-FLAG antibody, followed by western blotting with anti-flag and anti-M1 antibodies (lanes 4–6). The original blots are presented in Supplementary Fig. 9. (**f**) PRC2 complex facilitates the interaction of M1 with vRNP. Recombinant His-M1 (12 pmol) and vRNP purified from influenza virions were incubated without (lanes 1 and 4) or with either 8 pmol of 3xFLAG-EZH2 (lanes 2 and 5) or 8 pmol of 3xFLAG-SUZ12 (lanes 3 and 6) for 1 h. Then, vRNP was immunoprecipitated with anti-NP antibody, and then the co-immunoprecipitated M1 was detected by western blotting with anti-M1 antibody (lanes 4–6). The original blots are presented in Supplementary Fig. 10.

vRNP is exported from the nucleus to the cytoplasm mediated by the CRM1-dependent pathway through the interaction of M1 and NS2 with vRNP. To test whether PRC2 complex regulates the association of M1 with vRNP, we carried out proximity ligation assays (PLA) using rabbit anti-M1 and mouse anti-NP (mAb61A5) antibodies. It is reported that the mAb61A5 antibody recognizes NP on the viral genome^28^, and thus the interaction of M1 with vRNP can be detected by the *in situ* PLA system. The number of PLA signals between vRNP and M1 in EZH2 KD cells decreased to approximately 50% of that in control cells at 6 h post infection (Fig. 4a,b). These results suggest that PRC2 complex is involved in the interaction between M1 and vRNP.

### PRC2 complex facilitates M1-vRNP complex formation through the direct interaction with M1

To examine the molecular mechanism of the assembly of M1-vRNP complex stimulated by PRC2 complex, we performed immunoprecipitation assays using recombinant PRC2 complex and M1. 3xFLAG-EZH2 and SUZ12 were purified from 293T cells using anti-FLAG antibody-conjugated agarose (Fig. 4c, lanes 2 and 3, Supplementary Fig. 7). Other PRC2 subunits were also co-purified with 3xFLAG-EZH2 and 3xFLAG-SUZ12, respectively (Fig. 4d and Supplementary Fig. 8), which suggests that part of purified 3xFLAG-EZH2 and 3xFLAG-SUZ12 forms PRC2 complexes. We found that M1 was co-immunoprecipitated with PRC2 complexes purified with 3xFLAG-EZH2 and 3xFLAG-SUZ12, respectively (Fig. 4e, lanes 4–6 and Supplementary Fig. 9). Next, we tested the interaction of M1 with vRNP in the presence or absence of PRC2 complex by immunoprecipitation assays with anti-NP antibody using purified recombinant proteins. The amount of M1 co-immunoprecipitated with vRNP was increased in the presence of PRC2 complex (Fig. 4f, lanes 4–6 and Supplementary Fig. 10). These findings suggest that PRC2 complex could recruit M1 onto vRNA for the nuclear export of vRNP through the direct interaction of PRC2 complex with M1.

## Discussion

PRC2 complex has the methyltransferase activity of histone H3K27me3, which is one of the markers of fHC^29^. Recently, it was reported that CRM1 interacts with a particular focus of fHC, and recruits Nup98-HoxA9 and CALM-AF10 fusion proteins on fHC through the recognition of NES^30^,^31^. These findings suggest that NS2 could be recruited to CRM1-bound fHC through the NES. NP also binds to histone H3K27me3^11^, suggesting that vRNP localizes at fHC. Thus, it is possible that M1 is recruited to fHC through its interaction with PRC2 complex, and the export complex of vRNP is formed at fHC. CHD3, one of the deacetylase components of NuRD complex, also promotes the nuclear export of vRNP through its interaction with NS2^32^. It has been reported that NuRD complex binds to histone H3K27me3 and cooperates with PRC2 to suppress cellular gene expression^33^,^34^,^35^. Taken together, these findings suggest that fHC functions as an assembly site for the export complex of vRNP.

We demonstrated that EZH2 KD results in the accumulation of vRNP in the nucleus (Fig. 2a). Proteomics analyses revealed that a number of viral proteins are subjected to post-translational modifications for virus growth^36^. However, the treatment of GSK126 does not affect the localization of the viral genome (Fig. 2d). This suggests that the methyltransferase activity of PRC2 complex and histone H3K27me3 are not important for vRNP export. Previous reports showed that histone H3K27me3 is required for the assembly of heterochromatin, but not essential for the maintenance of the chromatin compaction^3^,^37^,^38^. Therefore, it is possible that the fHC region is important for the formation of vRNP export complex.

A number of different RNA and DNA viruses inhibit the formation of PML NB to prevent antiviral responses, and certain viruses hijack PML NB for their viral replication and transcription^39^,^40^. It is also reported that the proviral promoter of HIV is assembled at fHC near PML NB and that the promoter is activated in response to the disruption of PML NB^41^. Further, it is worthy to note that some parts of M1, NS2, and vRNA are found at PML NB^20^,^42^,^43^. Based on these reports, it is possible that PML NB is in close proximity to fHC, and that vRNP might translocate from PML NB to fHC. To further understand the regulatory mechanism of vRNP nuclear export, the significance of the accumulation of vRNP and viral proteins at PML NB requires further analysis.

## Methods

### Biological materials

Rabbit polyclonal antibodies against M1, PB1, PB2 NP and CTCF were prepared as previously described^13^,^44^,^45^,^46^. Rabbit polyclonal antibodies against EZH2 (Abcam), EED (Proteintech), SUZ12 (Proteintech), HDAC1 (Upstate), pol II (Santa Cruz), RAD21 (Abcam), and mouse monoclonal antibody against Brg1 (Santa Cruz), and rat polyclonal antibody against HP1β (Abcam) were purchased. Mouse monoclonal antibodies against NP (mAb61A5) and Topoisomerase II were a gift from Dr. F. Momose (Kitasato University)^28^ and Dr. A. Kikuchi (Nagoya University), respectively. vRNP was prepared from purified influenza virus (A/Puerto Rico/8/34) as previously described^47^. For the construction of the plasmids expressing 3xFLAG-EZH2 or 3xFLAG-SUZ12, cDNA was generated from total RNA extracted from HeLa cells using oligo dT primer (5′-TTTTTTTTTTTTTTTTTTTT-3′), then amplified with following specific primer sets: 5′-AGATCTACGCGTATGGGCCAGACTGGGAAGAAA-3′ and 5′-AGATCTACGCGTTCAAGGGATTTCCATTTCTCTTTCG-3′ for EZH2; 5′-CAGGAATTCCTCGAGATGGCGCCTCAGAAGCAC-3′ and 5′-CAGGAATTCCTCGAGTCAGAGTTTTTGTTTTTTGCTCTGT-3′ for SUZ12. To construct a plasmid for the expression of 3xFLAG-tagged proteins, the fragment 5′-GATCCGCCGCCACCATGGACTACAAGGATGACGACGACAAGGACTACAAGGATGACGACGACAAGGACTACAAGGATGACGACGACAAGCATATGCAGGAATTCGATATCAAGCTTATCGATACCGTCGACCTCGA-3′ was inserted to pCAGGS digested with *Eco*R I and filled with Klenow fragment. The amplified cDNAs of EZH2 and SUZ12 were cloned into pCAGGS-3xFLAG digested with *Eco*R I, respectively. HeLa cells (a kind gift from Dr. MA. Yamada, The University of Tokyo) and 293T cells (a kind gift from Dr. Y. Kawaoka, The University of Tokyo) were maintained in Dulbecco’s modified Eagle’s medium (DMEM) containing 10% fetal bovine serum (FBS), and MDCK cells (a kind gift from Dr. P. Palese, Icahn School of Medicine at Mount Sinai) were cultured in minimal essential medium (MEM) containing 10% FBS. GSK126 was purchased from Xcessbio.

### Immunoprecipitation

At 6 h post infection, cells were cross-linked with 0.5% of formaldehyde at room temperature for 10 min. Fixed cells were lysed by sonication in a lysis buffer containing 50 mM Tris-HCl (pH 7.9), 150 mM NaCl, and 0.1% Triton X-100. The lysates were centrifuged at 14,000x g, and the supernatants were subjected to the immunoprecipitation assays with antibodies as indicated in figure legends. After washing with lysis buffer, co-immunoprecipitated proteins were eluted with 0.1 M glycine (pH 3.0). Co-immunoprecipitated proteins were separated by SDS-PAGE and analyzed by western blotting with antibodies as indicated in each figure legend.

### Gene silencing mediated by siRNA

Cells (7 × 10^5^) were transfected with 30 pmol of either control siRNA or siRNA against EZH2 (Invitrogen) using lipofectamine RNAi Max (Life Technologies) according to the manufacture’s protocol.

### Quantification of viral RNA

Total RNAs were purified from infected cells using RNA purification kit (Nacalai). For RNP immunoprecipitation assays, infected cells were cross-linked with 0.5% of formaldehyde for 10 min at room temperature. Fixed cells were lysed by sonication in a buffer containing 12.5 mM Tris-HCl (pH 7.9), 150 mM NaCl, 5 μg/ml heparin, and 1% Triton X-100. The supernatants were recovered by centrifugation at 14,000x g for 10 min, and subjected to immunoprecipitation assays. After sequential washes with high salt buffer containing 20 mM Tris-HCl (pH 7.9), 2 mM EDTA, 500 mM NaCl, and 1% Triton X-100, and LiCl buffer containing 10 mM Tris-HCl (pH 7.9), 1 mM EDTA, 250 mM LiCl, 1% deoxycholate, and 1% NP-40, viral RNAs co-immunoprecipitated with either EZH2 or SUZ12 from infected cells were subjected to the quantitative real-time PCR as previously described^20^. vRNA and cRNA of segment 5 synthesized by T7 RNA polymerase *in vitro* (Promega) were used as a standard for quantitative RT-RCR to quantify the absolute amount.

### Indirect immunofluorescence assays and fluorescent *in situ* hybridization assays

Indirect immunofluorescence assays and fluorescent *in situ* hybridization assays were carried out as previously described^20^. Briefly, infected cells were fixed with 4% paraformaldehyde for 10 min at room temperature, permeabilized with PBS containing 0.5% Triton X-100 for 5 min at room temperature, and incubated with PBS containing 1% bovine serum albumin (BSA) for 1 h. Cells were incubated with primary antibodies as indicated in each figure legend for 1 h, and then incubated with Alexa Fluor488-conjugated secondary antibodies, respectively (Thermo Fisher Scientific). FISH assays were carried out using an RNA probe complementary to the segment 1 vRNA. Images were acquired as confocal sections taken at the same level of focus among samples.

### *In situ* proximity ligation assays (PLA)

*In situ* PLA was carried out as previously described^48^. Infected cells were fixed with 4% PFA for 10 min, followed by permeabilization with PBS containing 0.5% Triton X-100 at room temperature for 5 min. The cells were incubated with PBS containing 1% skim milk for 30 min, and incubated with rabbit anti-M1 and mouse anti-NP antibodies for 1 h. PLA assays were performed using Duolink *in situ* PLA kit (Olink Bioscience) according to the manufacturer’s protocol. Counting of the PLA foci was carried out using IMARIS software (Carl Zeiss).

### Purification of recombinant proteins

Purification of His-M1 was performed as previously described^49^. To purify the PRC2 complex, 293T cells were transfected with plasmids expressing either 3xFLAG-EZH2 or 3xFLAG-SUZ12, and then incubated for 48 h. Transfected cells were lysed by sonication in a buffer containing 20 mM Tris-HCl (pH 7.9), 100 mM NaCl, and 0.1% Triton X-100. Lysates were incubated with FLAG antibody-conjugated agarose (Sigma) at 4 °C for 1 h. After washing with a buffer (Wash buffer) containing 50 mM Tris-HCl (pH 7.9) and 175 mM KCl, 0.2 mM EDTA, 0.1% Triton X-100, and 10% glycerol, PRC2 complex was eluted with Wash buffer containing 0.5 mg/ml 3xFLAG peptide.

## Additional Information

**How to cite this article**: Asaka, M. N. *et al*. Polycomb repressive complex 2 facilitates the nuclear export of the influenza viral genome through the interaction with M1. *Sci. Rep*. **6**, 33608; doi: 10.1038/srep33608 (2016).

## Supplementary Material

Supplementary Information

## References

[b1] Josset L., Frobert E. & Rosa-Calatrava M. Influenza A replication and host nuclear compartments: many changes and many questions. J. Clin. Virol. 43, 381–390 (2008). 18926763 10.1016/j.jcv.2008.08.017

[b2] Black J. C. & Whetstine J. R. Chromatin landscape: methylation beyond transcription. Epigenetics 6, 9–15 (2011). 20855937 10.4161/epi.6.1.13331 PMC3052912

[b3] Cao R. . Role of histone H3 lysine 27 methylation in Polycomb-group silencing. Science 298, 1039–1043 (2002). 12351676 10.1126/science.1076997

[b4] Wang H. . Role of histone H2A ubiquitination in Polycomb silencing. Nature 431, 873–878 (2004). 15386022 10.1038/nature02985

[b5] Chan A. Y., Vreede F. T., Smith M., Engelhardt O. G. & Fodor E. Influenza virus inhibits RNA polymerase II elongation. Virology 351, 210–217 (2006). 16624367 10.1016/j.virol.2006.03.005

[b6] Marcos-Villar L., Pazo A. & Nieto A. Influenza virus and the chromatin: Role of CHD1 chromatin remodeler on virus life cycle. J. Virol. 90, 3694–3707 (2016). 26792750 10.1128/JVI.00053-16 PMC4794673

[b7] Engelhardt O. G., Smith M. & Fodor E. Association of the influenza A virus RNA-dependent RNA polymerase with cellular RNA polymerase II. J. Virol. 79, 5812–5818 (2005). 15827195 10.1128/JVI.79.9.5812-5818.2005 PMC1082766

[b8] Naito T. . An influenza virus replicon system in yeast identified Tat-SF1 as a stimulatory host factor for viral RNA synthesis. Proc. Natl. Acad. Sci. USA 104, 18235–18240 (2007). 17991777 10.1073/pnas.0705856104 PMC2084326

[b9] Garcia-Robles I., Akarsu H., Muller C. W., Ruigrok R. W. & Baudin F. Interaction of influenza virus proteins with nucleosomes. Virology 332, 329–336 (2005). 15661164 10.1016/j.virol.2004.09.036

[b10] Takizawa N., Watanabe K., Nouno K., Kobayashi N. & Nagata K. Association of functional influenza viral proteins and RNAs with nuclear chromatin and sub-chromatin structure. Microbes Infect. 8, 823–833 (2006). 16513387 10.1016/j.micinf.2005.10.005

[b11] Alfonso R. . CHD6 chromatin remodeler is a negative modulator of influenza virus replication that relocates to inactive chromatin upon infection. Cell Microbiol. 13, 1894–1906 (2011). 21899694 10.1111/j.1462-5822.2011.01679.x

[b12] Alfonso R., Rodriguez A., Rodriguez P., Lutz T. & Nieto A. CHD6, a cellular repressor of influenza virus replication, is degraded in human alveolar epithelial cells and mice lungs during infection. J. Virol. 87, 4534–4544 (2013). 23408615 10.1128/JVI.00554-12 PMC3624387

[b13] Watanabe K. . Inhibition of nuclear export of ribonucleoprotein complexes of influenza virus by leptomycin B. Virus Res. 77, 31–42 (2001). 11451485 10.1016/s0168-1702(01)00263-5

[b14] Elton D. . Interaction of the influenza virus nucleoprotein with the cellular CRM1-mediated nuclear export pathway. J. Virol. 75, 408–419 (2001). 11119609 10.1128/JVI.75.1.408-419.2001 PMC113933

[b15] Neumann G., Hughes M. T. & Kawaoka Y. Influenza A virus NS2 protein mediates vRNP nuclear export through NES-independent interaction with hCRM1. EMBO J. 19, 6751–6758 (2000). 11118210 10.1093/emboj/19.24.6751 PMC305902

[b16] Noton S. L. . Identification of the domains of the influenza A virus M1 matrix protein required for NP binding, oligomerization and incorporation into virions. J. Gen. Virol. 88, 2280–2290 (2007). 17622633 10.1099/vir.0.82809-0 PMC2884976

[b17] Baudin F., Petit I., Weissenhorn W. & Ruigrok R. W. *In vitro* dissection of the membrane and RNP binding activities of influenza virus M1 protein. Virology 281, 102–108 (2001). 11222100 10.1006/viro.2000.0804

[b18] O’Neill R. E., Talon J. & Palese P. The influenza virus NEP (NS2 protein) mediates the nuclear export of viral ribonucleoproteins. EMBO J. 17, 288–296 (1998). 9427762 10.1093/emboj/17.1.288 PMC1170379

[b19] Chase G. P. . Influenza virus ribonucleoprotein complexes gain preferential access to cellular export machinery through chromatin targeting. Plos Pathog. 7, e1002187 (2011). 21909257 10.1371/journal.ppat.1002187 PMC3164630

[b20] Kawaguchi A., Matsumoto K. & Nagata K. YB-1 functions as a porter to lead influenza virus ribonucleoprotein complexes to microtubules. J. Virol. 86, 11086–11095 (2012). 22855482 10.1128/JVI.00453-12 PMC3457152

[b21] Cao R. & Zhang Y. SUZ12 is required for both the histone methyltransferase activity and the silencing function of the EED-EZH2 complex. Mol. Cell 15, 57–67 (2004). 15225548 10.1016/j.molcel.2004.06.020

[b22] Jiao L. & Liu X. Structural basis of histone H3K27 trimethylation by an active polycomb repressive complex 2. Science 350, aac4383 (2015). 10.1126/science.aac4383 PMC5220110 26472914

[b23] Margueron R. . Role of the polycomb protein EED in the propagation of repressive histone marks. Nature 461, 762–767 (2009). 19767730 10.1038/nature08398 PMC3772642

[b24] Gjerstorff M. F. . SSX2 is a novel DNA-binding protein that antagonizes polycomb group body formation and gene repression. Nucleic Acids Res. 42, 11433–11446 (2014). 25249625 10.1093/nar/gku852 PMC4191419

[b25] Fiskus W. . Histone deacetylase inhibitors deplete enhancer of zeste 2 and associated polycomb repressive complex 2 proteins in human acute leukemia cells. Mol. Cancer Ther. 5, 3096–3104 (2006). 17172412 10.1158/1535-7163.MCT-06-0418

[b26] Birbach A. . Signaling molecules of the NF-kappa B pathway shuttle constitutively between cytoplasm and nucleus. J. Biol. Chem. 277, 10842–10851 (2002). 11801607 10.1074/jbc.M112475200

[b27] McCabe M. T. . EZH2 inhibition as a therapeutic strategy for lymphoma with EZH2-activating mutations. Nature 492, 108–112 (2012). 23051747 10.1038/nature11606

[b28] Momose F., Kikuchi Y., Komase K. & Morikawa Y. Visualization of microtubule-mediated transport of influenza viral progeny ribonucleoprotein. Microbes Infect. 9, 1422–1433 (2007). 17905627 10.1016/j.micinf.2007.07.007

[b29] Trojer P. & Reinberg D. Facultative heterochromatin: is there a distinctive molecular signature? Mol. Cell 28, 1–13 (2007). 17936700 10.1016/j.molcel.2007.09.011

[b30] Oka M. . Chromatin-prebound Crm1 recruits Nup98-HoxA9 fusion to induce aberrant expression of Hox cluster genes. Elife 5, e09540 (2016). 26740045 10.7554/eLife.09540 PMC4718815

[b31] Conway A. E., Haldeman J. M., Wechsler D. S. & Lavau C. P. A critical role for CRM1 in regulating HOXA gene transcription in CALM-AF10 leukemias. Leukemia 29, 423–432 (2015). 25027513 10.1038/leu.2014.221 PMC4297268

[b32] Hu Y. . CHD3 facilitates vRNP nuclear export by interacting with NES1 of influenza A virus NS2. Cell Mol. Life Sci. 72, 971–982 (2015). 25213355 10.1007/s00018-014-1726-9 PMC4323543

[b33] Reynolds N. . NuRD-mediated deacetylation of H3K27 facilitates recruitment of Polycomb Repressive Complex 2 to direct gene repression. EMBO J. 31, 593–605 (2012). 22139358 10.1038/emboj.2011.431 PMC3273378

[b34] Hu Y. . CHD3 protein recognizes and regulates methylated histone H3 lysines 4 and 27 over a subset of targets in the rice genome. Proc. Natl. Acad. Sci. USA 109, 5773–5778 (2012). 22451926 10.1073/pnas.1203148109 PMC3326492

[b35] Zhang H., Bishop B., Ringenberg W., Muir W. M. & Ogas J. The CHD3 remodeler PICKLE associates with genes enriched for trimethylation of histone H3 lysine 27. Plant Physiol. 159, 418–432 (2012). 22452853 10.1104/pp.112.194878 PMC3375975

[b36] Hutchinson E. C. . Mapping the phosphoproteome of influenza A and B viruses by mass spectrometry. Plos Pathog. 8, e1002993 (2012). 23144613 10.1371/journal.ppat.1002993 PMC3493474

[b37] Chandra T. . Independence of repressive histone marks and chromatin compaction during senescent heterochromatic layer formation. Mol. Cell 47, 203–214 (2012). 22795131 10.1016/j.molcel.2012.06.010 PMC3701408

[b38] Vallot C., Hérault A., Boyle S., Bickmore W. A. & Radvanyi F. PRC2-independent chromatin compaction and transcriptional repression in cancer. Oncogene 34, 741–751 (2015). 24469045 10.1038/onc.2013.604

[b39] Rivera-Molina Y. A., Martínez F. P. & Tang Q. Nuclear domain 10 of the viral aspect. World J. Virol. 2, 110–122 (2013). 24255882 10.5501/wjv.v2.i3.110 PMC3832855

[b40] Everett R. D. & Chelbi-Alix M. K. PML and PML nuclear bodies: implications in antiviral defence. Biochimie 89, 819–830 (2007). 17343971 10.1016/j.biochi.2007.01.004

[b41] Lusic M. . Proximity to PML nuclear bodies regulates HIV-1 latency in CD4+ T cells. Cell Host Microbe 13, 665–677 (2013). 23768491 10.1016/j.chom.2013.05.006

[b42] Sato Y. . Localization of influenza virus proteins to nuclear dot 10 structures in influenza virus-infected cells. Virology 310, 29–40 (2003). 12788628 10.1016/s0042-6822(03)00104-1

[b43] Shibata T., Tanaka T., Shimizu K., Hayakawa S. & Kuroda K. Immunofluorescence imaging of the influenza virus M1 protein is dependent on the fixation method. J. Virol. Methods 156, 162–165 (2009). 19027795 10.1016/j.jviromet.2008.10.020

[b44] Kawaguchi A., Momose F. & Nagata K. Replication-coupled and host factor-mediated encapsidation of the influenza virus genome by viral nucleoprotein. J. Virol. 85, 6197–6204 (2011). 21507964 10.1128/JVI.00277-11 PMC3126543

[b45] Naito T., Momose F., Kawaguchi A. & Nagata K. Involvement of Hsp90 in assembly and nuclear import of influenza virus RNA polymerase subunits. J. Virol. 81, 1339–1349 (2007). 17121807 10.1128/JVI.01917-06 PMC1797515

[b46] Komatsu T., Sekiya T. & Nagata K. DNA replication-dependent binding of CTCF plays a critical role in adenovirus genome functions. Sci. Rep. 3, 2187 (2013). 23851926 10.1038/srep02187 PMC3711053

[b47] Shimizu K., Handa H., Nakada S. & Nagata K. Regulation of influenza virus RNA polymerase activity by cellular and viral factors. Nucleic Acids Res. 22, 5047–5053 (1994). 7800498 10.1093/nar/22.23.5047 PMC523776

[b48] Kumakura M., Kawaguchi A. & Nagata K. Actin-myosin network is required for proper assembly of influenza virus particles. Virology 476, 141–150 (2015). 25543965 10.1016/j.virol.2014.12.016

[b49] Watanabe K., Handa H., Mizumoto K. & Nagata K. Mechanism for inhibition of influenza virus RNA polymerase activity by matrix protein. J. Virol. 70, 241–247 (1996). 8523532 10.1128/jvi.70.1.241-247.1996 PMC189810

