# Supplementary material for: Polycomb repressive complex 2 facilitates the nuclear export of the influenza viral genome through the interaction with M1
Source: Sci Rep. 2016 Sep 20;6:33608. doi: 10.1038/srep33608 (PMC5028886; doi:10.1038/srep33608)
Supplement: Supplementary Information [file srep33608-s1.pdf]

Title: Polycomb repressive complex 2 facilitates the nuclear export of the influenza viral genome through the interaction with M1

Authors: Masamitsu N Asaka[1], Atsushi Kawaguchi[1*], Yuri Sakai[2], Kotaro Mori[1], and Kyosuke Nagata[1*]

Addresses:

[1]Department of Infection Biology, Faculty of Medicine and Graduate School of Comprehensive Human Sciences, University of Tsukuba, Tsukuba, Japan

[2]Ph.D. Program in Human Biology, School of Integrative and Global Majors, University of Tsukuba, Tsukuba, Japan

*Correspondence should be addressed to K. N. (email: knagata@md.tsukuba.ac.jp), and A. K. (email: ats-kawaguchi@md.tsukuba.ac.jp).

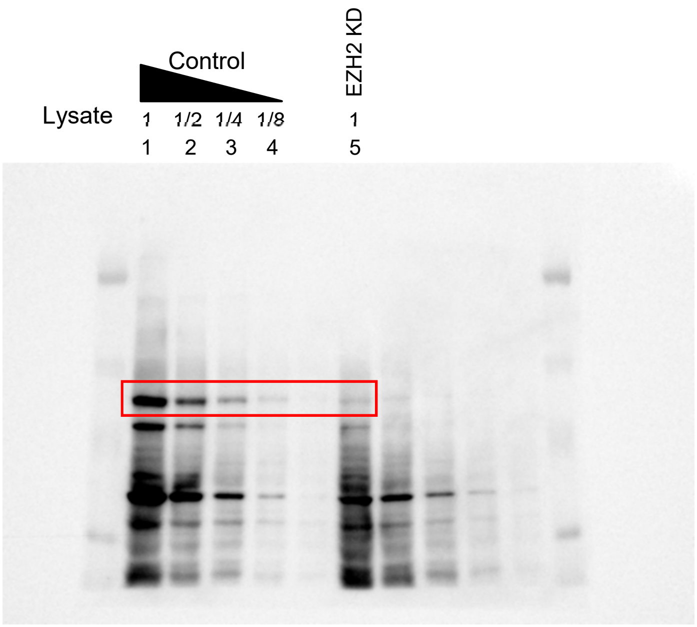

Supplementary Figure 1

The original blot of Figure 1b. HeLa cells were transfected with either control siRNA or siRNA against EZH2. At 60 h post transfection, 1 x 10^5 cells (lane 1), 5 x 10^4 cells (lane 2), 2.5 x 10^4 cells (lane 3), 1.25 x 10^4 cells (lane 4) of control cells, and 1 x 10^5 cells of EZH2 KD cells (lane 5) were subjected western blotting with anti-EZH2 antibody.

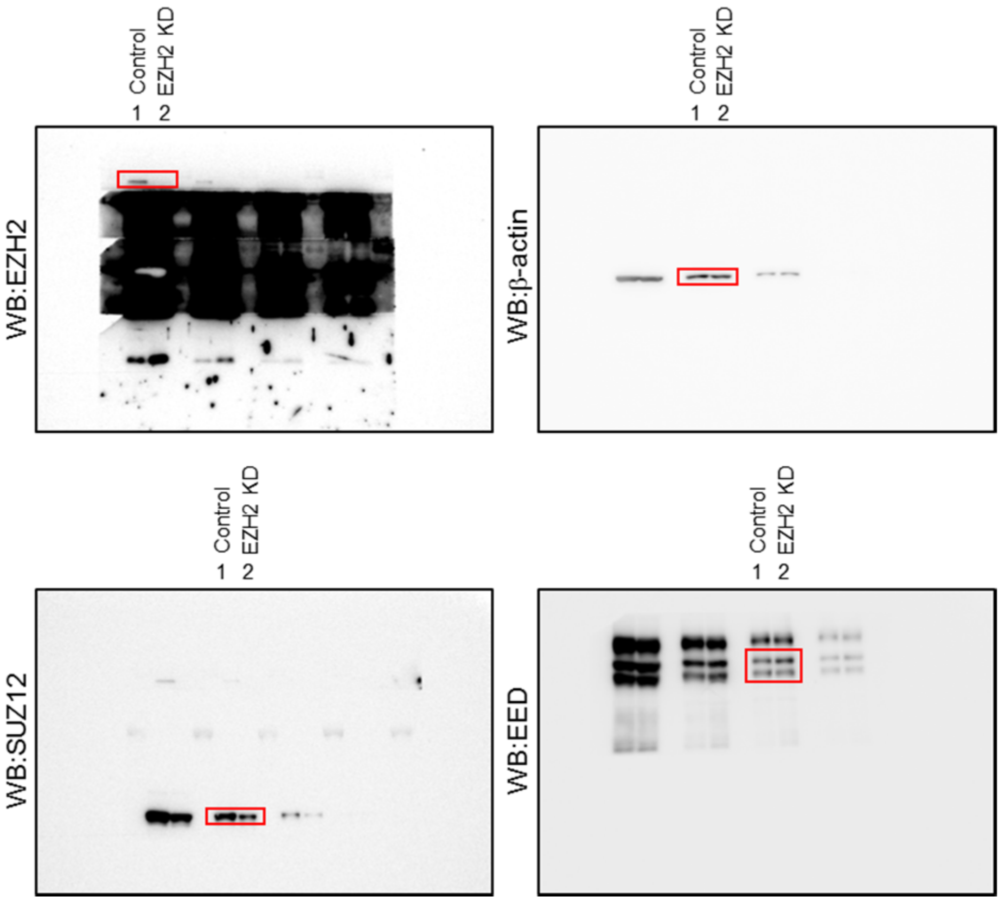

Supplementary Figure 2

The original blots of Figure 1c. HeLa cells were transfected with either control siRNA or siRNA against EZH2. At 60 h post transfection, control cells (lane 1), and EZH2 KD cells (lane 2) were subjected to western blotting with anti-EZH2, anti-SUZ12, anti-EED, and anti-β-actin antibodies.

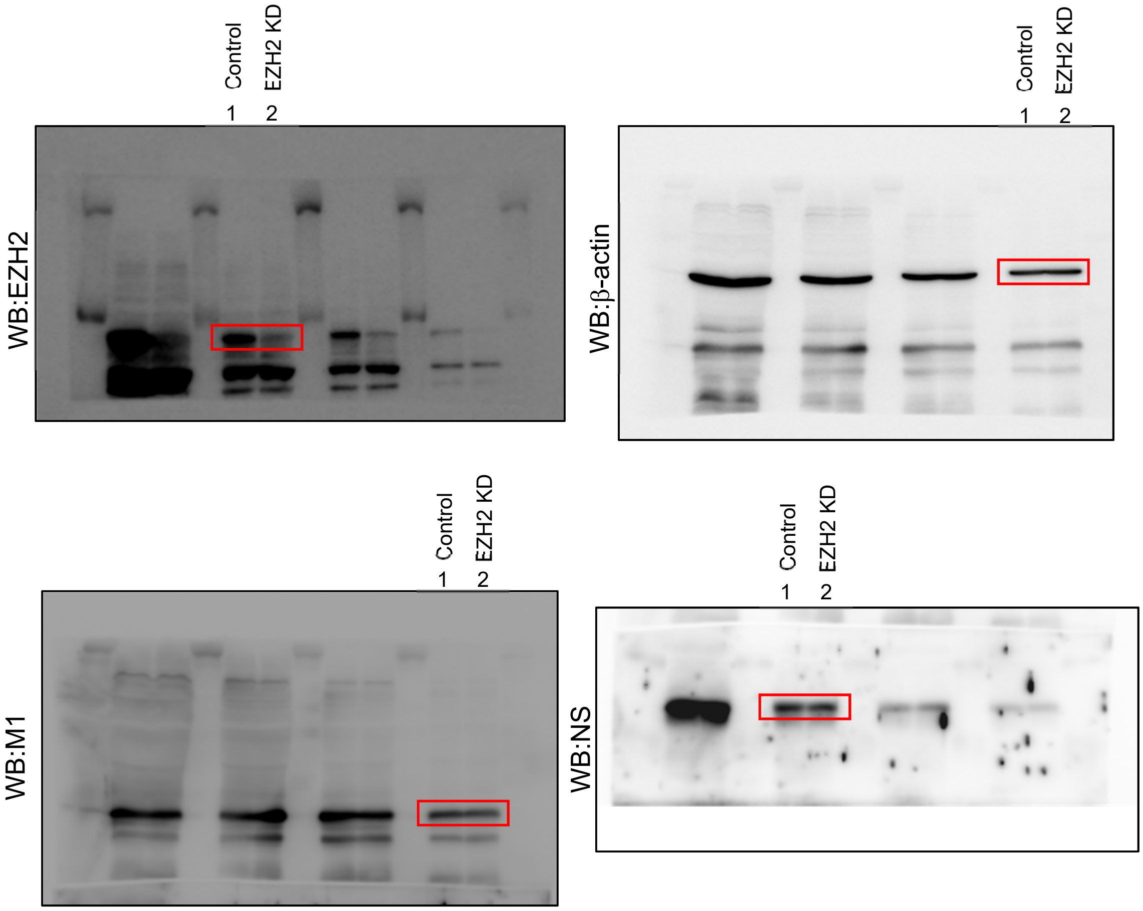

Supplementary Figure 3

The original blots of Figure 1f. HeLa cells were transfected with either control siRNA or siRNA against EZH2. At 60 h post transfection, control and EZH2 KD cells were infected with influenza virus at MOI of 5. At 6 h post infection, control and EZH2 KD cells were subjected to SDS-PAGE, and carried out western blotting with anti-EZH2, anti-M1, anti-NS2, and anti-β-actin antibodies.

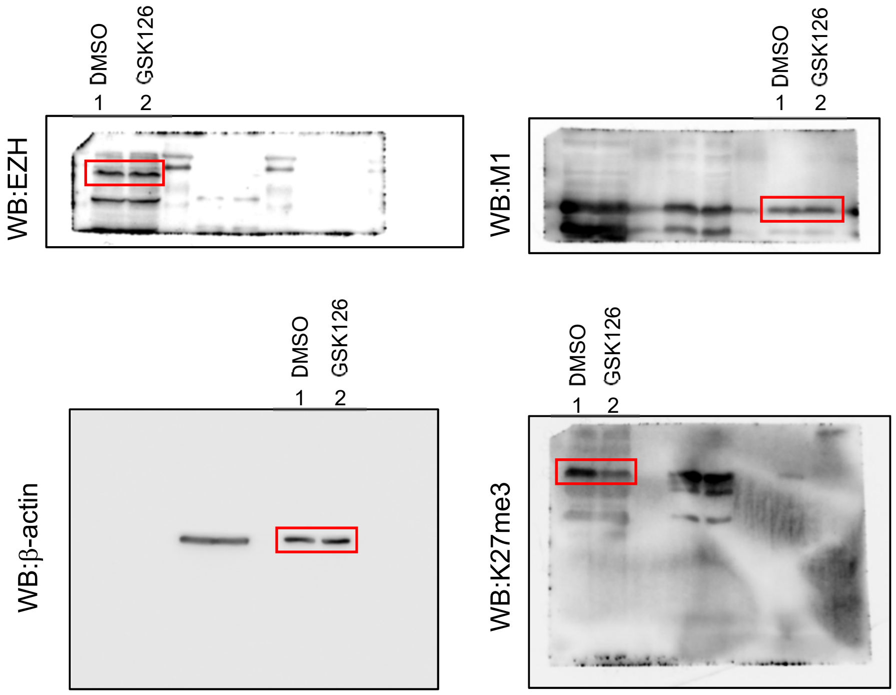

Supplementary Figure 4

The original blots of Figure 2c. HeLa cells were treated with either DMSO or 500 nM of GSK126 for 60 h. The cells were infected with influenza virus at MOI of 5. At 6 h post infection, cells were subjected to western blotting with anti-EZH2, anti-histone H3K27me3, anti-M1, and anti-β-actin antibodies.

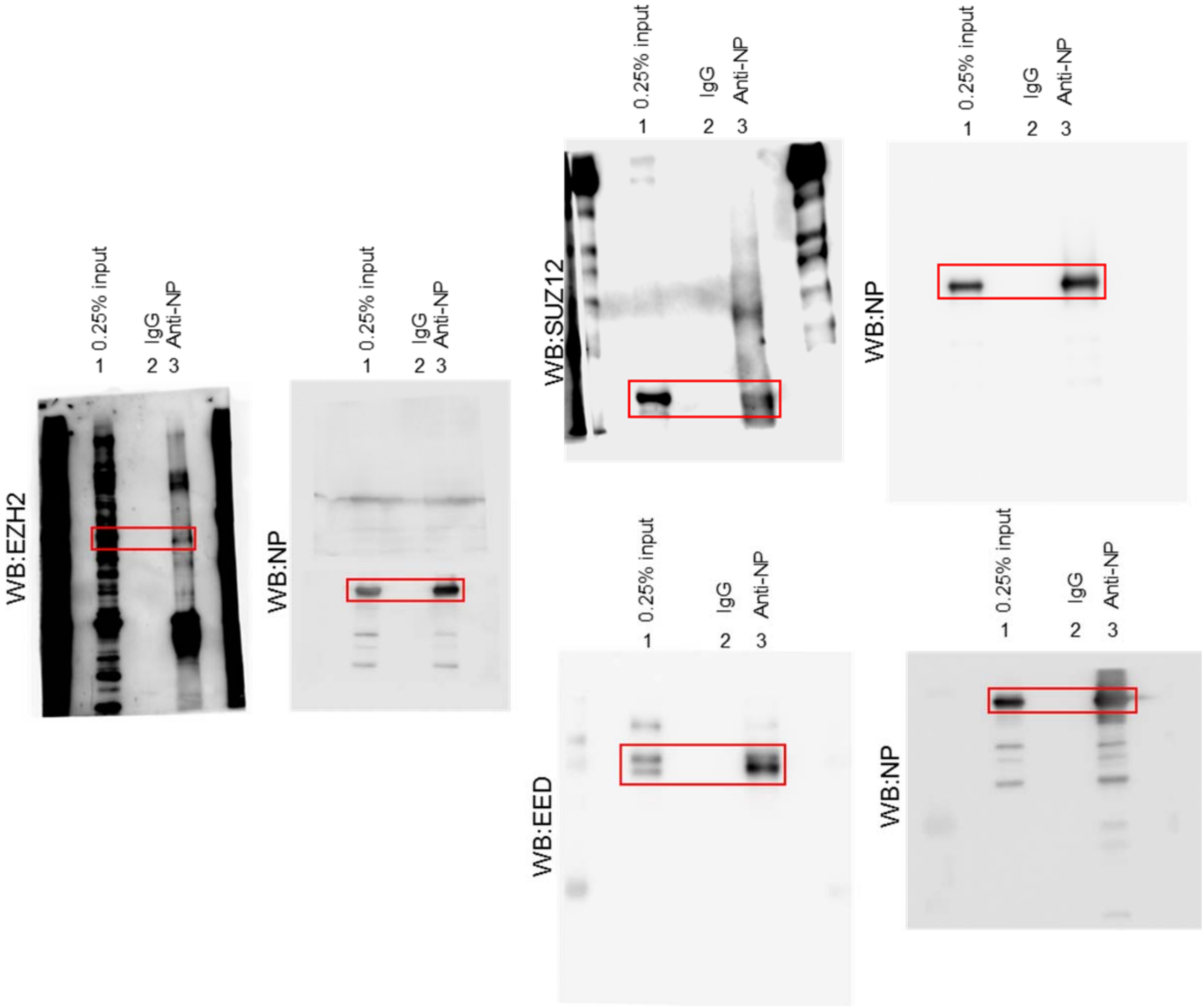

Supplementary Figure 5

The original blots of Figure 3a. At 6 h post infection, infected cells were subjected to immunoprecipitation assays with either non-specific IgG (lane 2) or anti-NP (lane 3) antibody-conjugated protein A Sepharose. Co-immunoprecipitated proteins were separated by 10% of SDS-PAGE, and analyzed by western blotting with anti-EZH2, anti-EED, anti-SUZ12, and anti-NP antibodies.

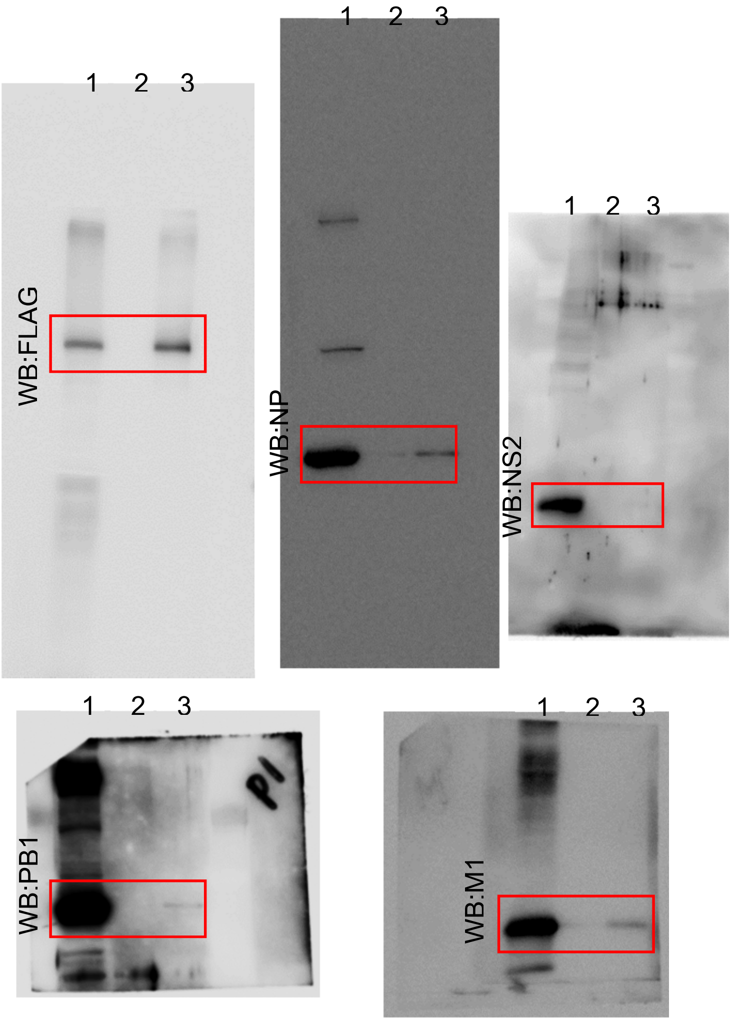

Supplementary Figure 6

The original blots of Figure 3c. HeLa cells were transfected with a plasmid expressing 3xFLAG-EZH2, and then infected with influenza virus at MOI of 5. At 6 h post infection, these cells were subjected to immunoprecipitation assays with either non-specific IgG (lane 2) or anti-FLAG (lane3) antibody-conjugated Sepharose. Co-immunoprecipitated proteins were separated by 7.5% or 12.5% of SDS-PAGE, and analyzed by western blotting with anti-FLAG, anti-PB1, anti-PB2, anti-NP, anti-M1, and anti-NS2 antibodies.

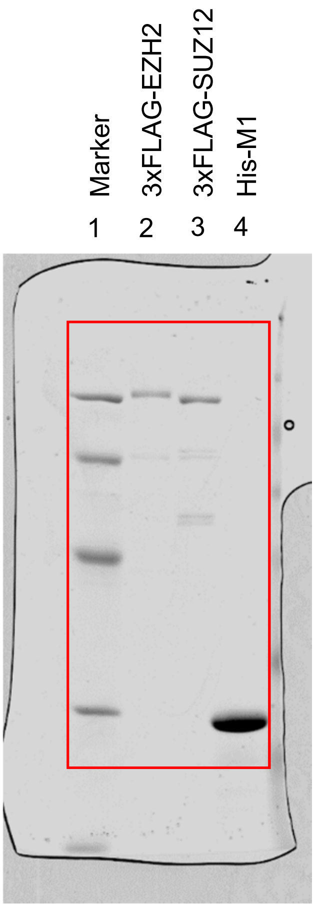

Supplementary Figure 7

The original blot of Figure 4c. Purified 3xFLAG-EZH2, 3xFLAG-SUZ12, and His-M1 proteins were applied to 10% SDS-PAGE, and visualized by CBB staining.

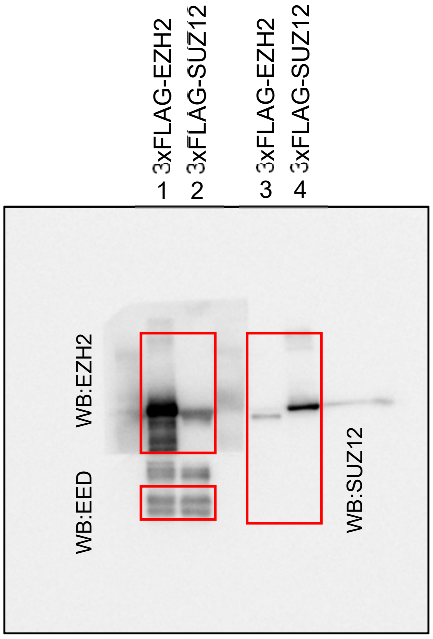

Supplementary Figure 8

The original blot of Figure 4d. Purified proteins were subjected to western blotting with anti-EZH2, anti-SUZ12, and anti-EED antibodies.

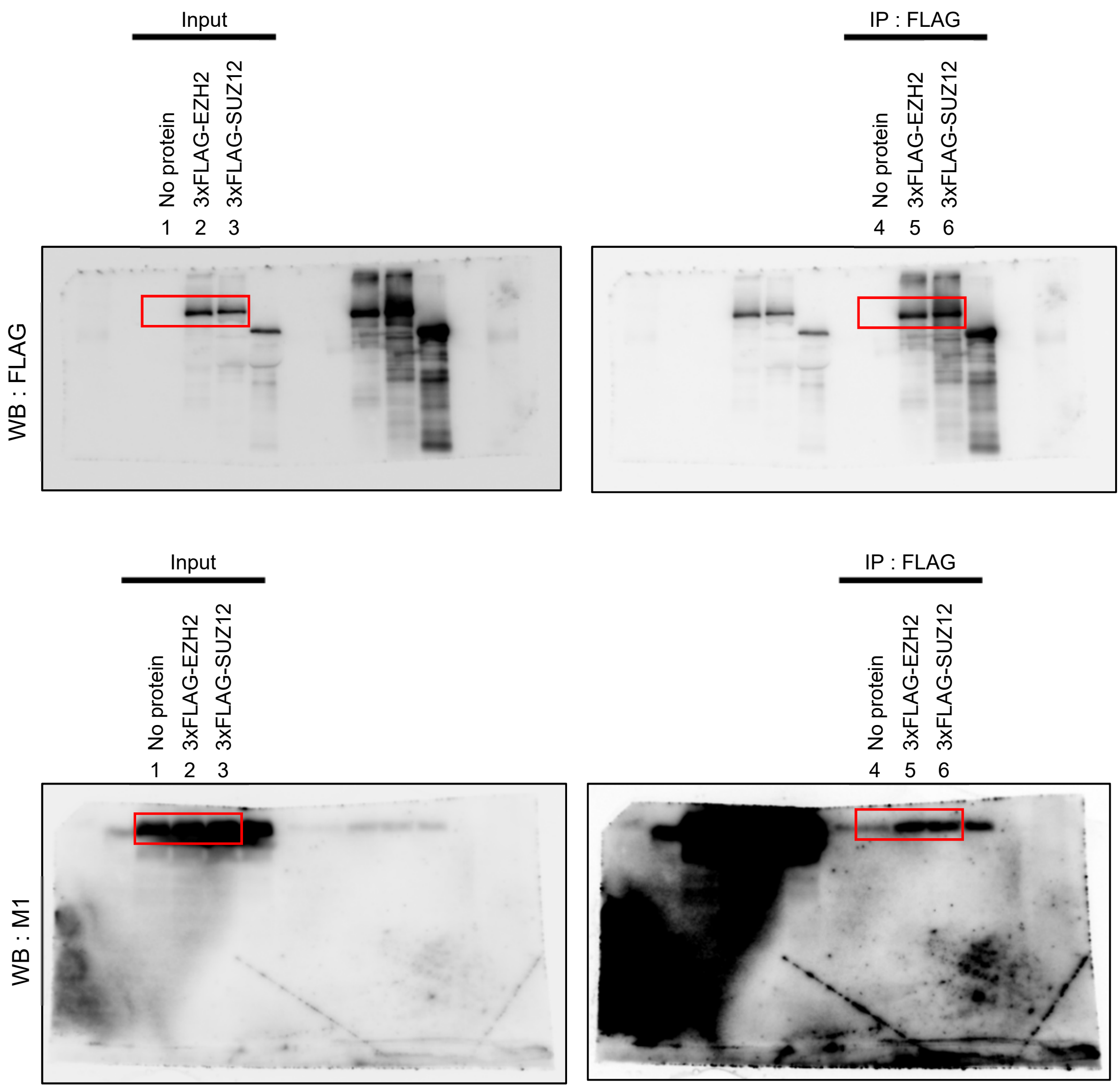

Supplementary Figure 9

The original blots of Figure 4e. Recombinant His-M1 (12 pmol) was incubated without (lanes 1 and 4) or with either 8 pmol of 3xFLAG-EZH2 (lanes 2 and 5) or 8 pmol of 3xFLAG-SUZ12 (lanes 3 and 6) for 1 h. Then, the proteins were subjected to immunoprecipitation assays with anti-FLAG antibody, followed by western blotting with anti-flag and anti-M1 antibodies (lanes 4-6).

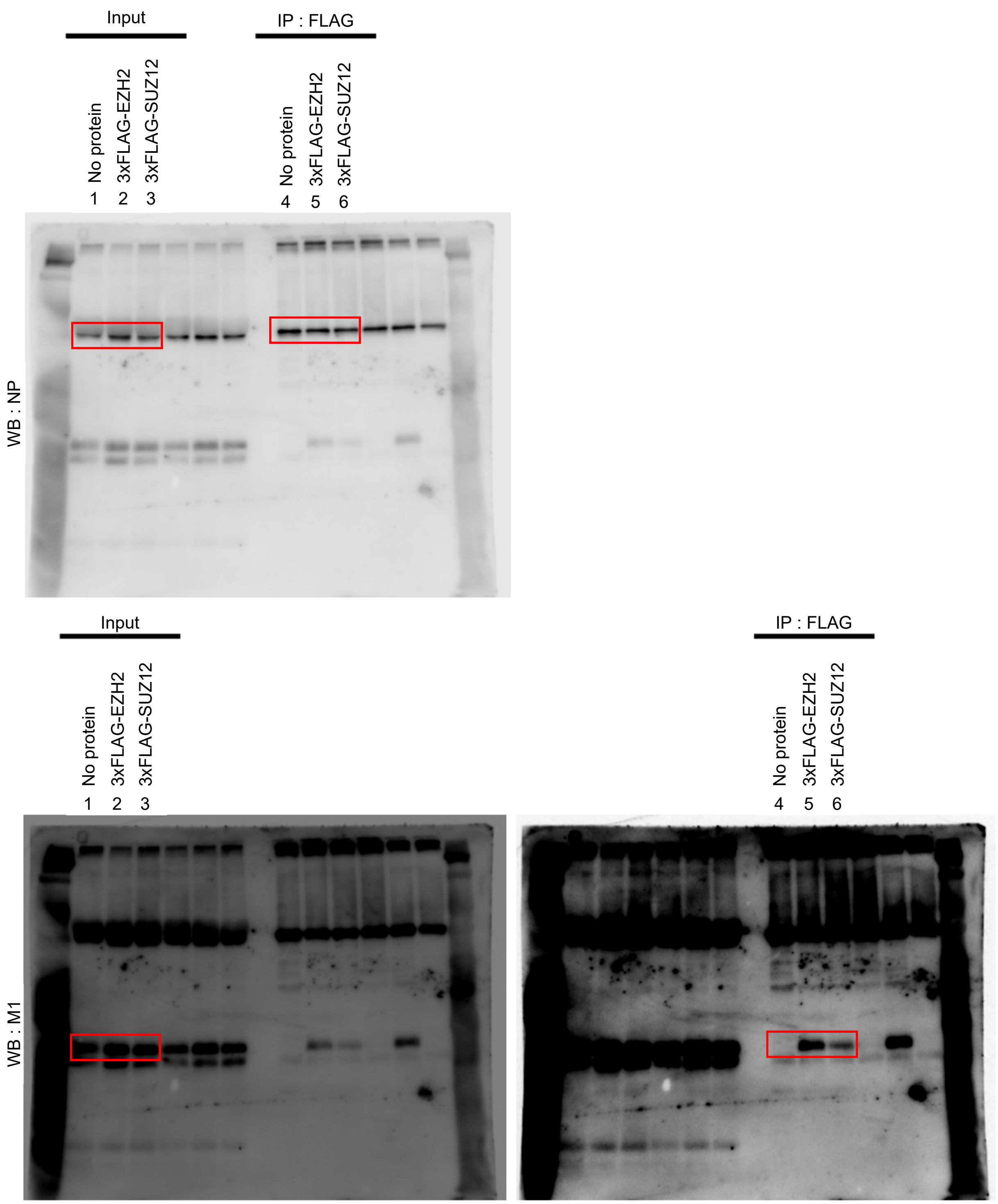

Supplementary Figure 10

The original blots of Figure 4f. Recombinant His-M1 (12 pmol) and vRNP purified

from influenza virions were incubated without (lanes 1 and 4) or with either 8 pmol of 3xFLAG-EZH2 (lanes 2 and 5) or 8 pmol of 3xFLAG-SUZ12 (lanes 3 and 6) for 1 h. Then, vRNP was immunoprecipitated with anti-NP antibody, and then the co-immunoprecipitated M1 was detected by western blotting with anti-M1 antibody (lanes 4-6).
